# What is e-health?

**DOI:** 10.2196/jmir.3.2.e20

**Published:** 2001-06-18

**Authors:** G Eysenbach

## Introduction

Everybody talks about e-health these days, but few people have come up with a clear definition of this comparatively new term. Barely in use before 1999, this term now seems to serve as a general "buzzword," used to characterize not only "Internet medicine", but also virtually everything related to computers and medicine. The term was apparently first used by industry leaders and marketing people rather than academics. They created and used this term in line with other "e-words" such as e-commerce, e-business, e-solutions, and so on, in an attempt to convey the promises, principles, excitement (and hype) around e-commerce (electronic commerce) to the health arena, and to give an account of the new possibilities the Internet is opening up to the area of health care. Intel, for example, referred to e-health as "a concerted effort undertaken by leaders in health care and hi-tech industries to fully harness the benefits available through convergence of the Internet and health care." Because the Internet created new opportunities and challenges to the traditional health care information technology industry, the use of a new term to address these issues seemed appropriate. These "new" challenges for the health care information technology industry were mainly (1) the capability of consumers to interact with their systems online (B2C = "business to consumer"); (2) improved possibilities for institution-to-institution transmissions of data (B2B = "business to business"); (3) new possibilities for peer-to-peer communication of consumers (C2C = "consumer to consumer").

So, how can we define e-health in the academic environment? One JMIR Editorial Board member feels that the term should remain in the realm of the business and marketing sector and should be avoided in scientific medical literature and discourse. However, the term has already entered the scientific literature (today, 76 Medline-indexed articles contain the term "e-health" in the title or abstract). What remains to be done is - in good scholarly tradition - to define as well as possible what we are talking about. However, as another member of the Editorial Board noted, "stamping a definition on something like e-health is somewhat like stamping a definition on 'the Internet': It is defined how it is used - the definition cannot be pinned down, as it is a dynamic environment, constantly moving."

It seems quite clear that e-health encompasses more than a mere technological development. I would define the term and concept as follows:

e-health is an emerging field in the intersection of medical informatics, public health and business, referring to health services and information delivered or enhanced through the Internet and related technologies. In a broader sense, the term characterizes not only a technical development, but also a state-of-mind, a way of thinking, an attitude, and a commitment for networked, global thinking, to improve health care locally, regionally, and worldwide by using information and communication technology.

This definition hopefully is broad enough to apply to a dynamic environment such as the Internet and at the same time acknowledges that e-health encompasses more than just "Internet and Medicine".

As such, the "e" in e-health does not only stand for "electronic," but implies a number of other "e's," which together perhaps best characterize what e-health is all about (or what it *should* be). Last, but not least, all of these have been (or will be) issues addressed in articles published in the Journal of Medical Internet Research.

## The 10 e's in "e-health"

1. **Efficiency** - one of the promises of e-health is to increase efficiency in health care, thereby decreasing costs. One possible way of decreasing costs would be by avoiding duplicative or unnecessary diagnostic or therapeutic interventions, through enhanced communication possibilities between health care establishments, and through patient involvement.
2. **Enhancing quality** of care - increasing efficiency involves not only reducing costs, but at the same time improving quality. E-health may enhance the quality of health care for example by allowing comparisons between different providers, involving consumers as additional power for quality assurance, and directing patient streams to the best quality providers.
3. **Evidence based** - e-health interventions should be evidence-based in a sense that their effectiveness and efficiency should not be assumed but proven by rigorous scientific evaluation. Much work still has to be done in this area.
4. **Empowerment** of consumers and patients - by making the knowledge bases of medicine and personal electronic records accessible to consumers over the Internet, e-health opens new avenues for patient-centered medicine, and enables evidence-based patient choice.
5. **Encouragement** of a new relationship between the patient and health professional, towards a true partnership, where decisions are made in a shared manner.
6. **Education** of physicians through online sources (continuing medical education) and consumers (health education, tailored preventive information for consumers)
7. **Enabling** information exchange and communication in a standardized way between health care establishments.
8. **Extending** the scope of health care beyond its conventional boundaries. This is meant in both a geographical sense as well as in a conceptual sense. e-health enables consumers to easily obtain health services online from global providers. These services can range from simple advice to more complex interventions or products such a pharmaceuticals.
9. **Ethics** - e-health involves new forms of patient-physician interaction and poses new challenges and threats to ethical issues such as online professional practice, informed consent, privacy and equity issues.
10. **Equity** - to make health care more equitable is one of the promises of e-health, but at the same time there is a considerable threat that e-health may deepen the gap between the "haves" and "have-nots". People, who do not have the money, skills, and access to computers and networks, cannot use computers effectively. As a result, these patient populations (which would actually benefit the most from health information) are those who are the least likely to benefit from advances in information technology, unless political measures ensure equitable access for all. The digital divide currently runs between rural vs. urban populations, rich vs. poor, young vs. old, male vs. female people, and between neglected/rare vs. common diseases.

In addition to these 10 essential e's, e-health should also be

- easy-to-use,
- entertaining (no-one will use something that is boring!) and
- exciting

- and it should definitely exist!

We invite other views on the definition of e-health and hope that over time the journal will be filled with articles which together elucidate the realm of e-health.

*Gunther Eysenbach*

*Editor,*

*Journal of Medical Internet Research*

